# Cervical Microbiota in Women with Preterm Prelabor Rupture of Membranes

**DOI:** 10.1371/journal.pone.0126884

**Published:** 2015-05-20

**Authors:** Marian Kacerovsky, Filip Vrbacky, Radka Kutova, Lenka Pliskova, Ctirad Andrys, Ivana Musilova, Ramkumar Menon, Ronald Lamont, Jana Nekvindova

**Affiliations:** 1 Biomedical Research Center, University Hospital Hradec Kralove, Hradec Kralove, Czech Republic; 2 Department of Obstetrics and Gynecology, Charles University in Prague, Faculty of Medicine Hradec Kralove, Hradec Kralove, Czech Republic; 3 4^th^ Department of Medicine, Hematology, University Hospital Hradec Kralove and Charles University in Prague, Faculty of Medicine in Hradec Kralove, Hradec Kralove, Czech Republic; 4 Institute of Clinical Biochemistry and Diagnostics, University Hospital Hradec Kralove, Hradec Kralove, Czech Republic; 5 Department of Clinical Immunology and Allergology, University Hospital Hradec Kralove, Charles University in Prague, Hradec Kralove, Czech Republic; 6 Department of Obstetrics and Gynecology, Division of Maternal-Fetal Medicine & Perinatal Research, The University of Texas Medical Branch at Galveston, Galveston, Texas, United States of America; 7 Department of Obstetrics and Gynecology, University of Southern Denmark, Odense University Hospital, Odense, Denmark; Xavier Bichat Medical School, INSERM-CNRS - Université Paris Diderot, FRANCE

## Abstract

**Objective:**

To analyze the cervical microbiota in women with preterm prelabor rupture of membranes (PPROM) by pyrosequencing and to document associations between cervical microbiota, cervical inflammatory response, microbial invasion of the amniotic cavity (MIAC), histological chorioamnionitis, and intraamniotic infection (IAI).

**Study Design:**

Sixty-one women with singleton pregnancies complicated by PPROM were included in the study. Specimens of cervical and amniotic fluid were collected on admission. The cervical microbiota was assessed by 16S rRNA gene sequencing by pyrosequencing. Interleukin (IL)-6 concentration in the cervical fluid and amniotic fluid was measured by ELISA and lateral flow immunoassay, respectively.

**Results:**

Four bacterial community state types [CST I (*Lactobacillus crispatus* dominated), CST III (*Lactobacillus iners* dominated), CST IV-A (non-*Lactobacillus* bacteria dominated), and CST IV-B (*Gardnerella vaginalis* and *Sneathia sanguinegens* dominated)] were observed in the cervical microbiota of women with PPROM. Cervical fluid IL-6 concentrations differed between CSTs (CST I = 145 pg/mL, CST III = 166 pg/mL, CST IV-A = 420 pg/mL, and CST IV-B = 322 pg/mL; *p* = 0.004). There were also differences in the rates of MIAC, of both MIAC and histological chorioamnionitis, and of IAI among CSTs. No difference in the rate of histological chorioamnionitis was found among CSTs.

**Conclusions:**

The cervical microbiota in PPROM women in this study was characterized by four CSTs. The presence of non-*Lactobacillus* CSTs was associated with a strong cervical inflammatory response and higher rates of MIAC, both MIAC and histological chorioamnionitis, and IAI representing a PPROM subtype with pronounced inflammation. CST I represents the dominant type of PPROM with a low rate of MIAC, IAI, and the combination of MIAC and histological chorioamnionitis.

## Introduction

Preterm prelabor rupture of membranes (PPROM), is defined as rupture of the fetal membranes with leakage of amniotic fluid (AF) before the onset of regular uterine contractions before 37 completed weeks of gestation, and represents a serious perinatal problem [1,2]. PPROM is responsible for approximately 3% of all births and 30% of all preterm births [1,2]. PPROM remains an unpredictable complication of pregnancy [3] and appears to be a disease of the fetal membranes characterized by premature aging where senescence, apoptosis and proteolysis play an important role in its pathophysiology [4–6]. Despite the fact that PPROM may have a non-infectious etiology, approximately 30% of cases are associated with microbial invasion of the amniotic cavity (MIAC) with *Ureaplasma* species being the most common bacteria isolated, and following PPROM, 60–70% will have evidence of histological chorioamnionitis [7–10]. PPROM pregnancies are often characterized by thinning of the chorion and bacterial colonization of the fetal membranes compared to uncomplicated pregnancies or pregnancies with spontaneous preterm labor with intact membranes [11]. Abnormal lower genital tract flora or viral infection of the cervix may predispose to ascending infection, microbial adhesion, colonization and infiltration of the choriodecidual space and fetal membranes leading to MIAC [12]. The subsequent host inflammatory response to MIAC can be detrimental to the fetus through the fetal inflammatory response syndrome [13,14].

Despite the increasing number of published microbiome studies, most of our knowledge of the composition of lower genital tract microbial flora comes from qualitative and semi-quantitative studies using culture-dependent techniques [15–18]. The development of culture-independent, molecular techniques such as those based on 16S rRNA gene sequencing has provided new information about the composition of normal and abnormal lower genital tract flora [19]. Those studies have shown that: i) communities of lower genital tract microflora differ between women; ii) they are species rich; iii) lower genital flora is strongly influenced by ethnicity and race; iv) different sites of sampling (lower or upper vagina) produce different results and influence the robustness of the measurements and v) bacteria that have not been detected by culture-based methods play a pivotal role in determining pregnancy outcome [20–25]. Based on 16S rDNA sequencing, six community state types (CSTs) of lower genital tract microbiota have been established [20,21]. Four of these CSTs are *Lactobacillus* dominated (CST I—*Lactobacillus crispatus*; CST II—*Lactobacillus gasseri*; CST III—*Lactobacillus iners* and CST V—*Lactobacillus jensenii*) [20,21]. The remaining two CSTs (CST IV-A and IV-B) are non-*Lactobacillus* bacteria dominated, with CST IV-B characterized by bacteria that are typically associated with bacterial vaginosis (BV) [20,21].

Recent reports by Romero et al. have shown that the lower genital tract microbiota of women changes during healthy pregnancy but is more stable than in non-pregnant women [22,26]. Moreover, the composition of lower genital tract microbiota is not different between women who had a spontaneous preterm delivery before 34 completed weeks of gestation and women who delivered at term [26]. On the other hand, Hyman et al. has shown that lower genital tract microbiota in Caucasian women differs between those who delivered preterm and at term [24].

There is a paucity of information pertaining to lower genital tract microbiota in pregnancies complicated by PPROM and its association with MIAC. Accordingly, the primary objective of this study was to characterize the cervical microbiota in women following PPROM by sequencing the 16S rDNA using high-throughput technology. We also wanted to evaluate the cervical inflammatory response, determined by interleukin (IL)-6 concentrations, and its relationship with the cervical microbiota. In addition, we evaluated whether there was an association between the cervical microbiota and MIAC with or without histological chorioamnionitis and intraamniotic infection (IAI). The objectives were derived from our overarching hypothesis that the varied microbiome signature in PPROM will correlate with inflammatory profile irrespective of the influence of potential AF antimicrobials and inflammatory markers. Additionally, we postulate that the microbiome-inflammatory biomarker pattern can be used to classify different subsets of PPROM to better understand the underlying pathology.

## Material and Methods

The Institutional Review Board at University Hospital Hradec Kralove (August 10, 2010; No. 201012 S15P) approved the study and written informed consent was obtained from all the participants. Between June 2011 and December 2012, a prospective cohort study was conducted. Women aged ≥18 years with a singleton pregnancy complicated by PPROM between 24 and 36 completed weeks of gestation who were admitted to the Department of Obstetrics and Gynecology, University Hospital Hradec Kralove, Czech Republic, were recruited. Exclusion criteria were: signs of intrauterine fetal growth restriction, vaginal bleeding, structural and/or chromosomal abnormalities of the fetus, signs of fetal hypoxia or any medical complication such as hypertension, preeclampsia, diabetes mellitus or thyroid disease. Gestational age was established based on the first trimester ultrasound scan for all pregnancies.

PPROM was defined as leakage of AF for at least two hours prior to the onset of labor. The diagnosis of PPROM was made using a sterile speculum examination by visualizing the characteristic pooling of AF in the vagina, together with a positive test for the presence of insulin-like growth factor–binding protein (ACTIM PROM test; Medix Biochemica, Kauniainen, Finland) in vaginal fluid. The management of PPROM in the Czech Republic is active (except for pregnancies before 28 completed weeks of gestation) and occurs no later than 72 hours after spontaneous rupture of the membranes. Either labor is induced or elective caesarean section is performed depending on the gestational age (within 24 hours for gestational ages > 34 weeks, within 48 hours for gestational ages between 32 and 34 weeks, and within 72 hours for gestational ages between 28 and 31 weeks), fetal wellbeing, maternal serum C-reactive protein levels, and cervicovaginal Group B Streptococcus colonization. AF and cervical fluid samples were collected at the same time in all women on admission, prior to the administration of corticosteroids, antibiotics, or tocolytics.

### AF and cervical fluid sampling

Ultrasound-guided transabdominal amniocentesis was performed and approximately 5mL of AF was aspirated and transported to the laboratory for DNA isolation, PCR detection of *Ureaplasma* species, *Mycoplasma hominis*, *Chlamydia trachomatis*, and 16S rRNA sequencing. Cervical fluid was obtained using a Dacron polyester swab, which was placed in the cervical canal for 20 seconds to achieve saturation and then inserted into polypropylene tubes containing 1.5mL of phosphate buffered saline. The tube was shaken for 20 minutes followed by centrifugation for 15 minutes at 300x *g* at room temperature. The supernatant was aliquoted and stored at -70°C for analysis of IL-6 concentration and the pellet was used for microbiota analysis.

### Detection of bacteria in the cervical fluid

Eubacterial 16S rDNA region V4-V6 was amplified using a slightly modified protocol of Baldrian et al., (2012) [27]. Briefly, two 200μl aliquot samples of the supernatant were used for parallel DNA isolation using QIAamp DNA Mini Kit (Qiagen, Netherlands) to ensure balanced sampling and extraction of the DNA. The isolation was performed according to manufacturer’s instructions with an elution volume of 200μl.

The PCR reaction (30μl) contained 12 pmol of each primer (eub530F/eub1100R), 6 nmol dNTPs, 0.9μl of enzyme mix (4% Pfu DNA Polymerase in DyNAzyme II DNA polymerase) (both Thermo Fisher Scientific, USA) and 100ng DNA in 1X DyNAzyme DNA Polymerase buffer. The PCR thermal profile was as follows: initial denaturation at 94°C for 5 min; 35 cycles of 94°C for 1 min, 55°C for 1 min and 72°C for 1 min, final extension at 72°C for 10 min. Each of the two DNA isolates was first preamplified in two parallel PCR reactions to reduce amplification bias. All four PCR products were pooled, checked by agarose electrophoresis and purified using Wizard SV Gel and PCR Clean-up System (Promega, USA) according to manufacturer’s protocol with elution in 20μl. The second PCR amplification was done with tag-encoded pyrosequencing primers (HPLC purified) containing sample tags separated from 16S primers by spacers and Titanium A or B adaptors (Roche, Switzerland). Spacer sequences were designed to contain a trinucleotide, absent in all GenBank sequences at this position to avoid preferential amplification of some targets [28]. The PCR protocol was the same as in the preamplification except that fusion primers were used and cycle number was 10. PCR products were extracted from electrophoretic gel using Wizard SV Gel and PCR Clean-Up System (Promega, USA) and purified using MinElute PCR Purification Kit (Qiagen, Netherlands) with elution in 10 μl. DNA was quantified using Quant-iT PicoGreen dsDNA Assay (Thermo Fisher Scientific, USA) and equimolar amounts of the PCR products were pooled together. The mixture was subjected to sequencing on a GS FLX+ sequencer (Roche, Switzerland) using GS Titanium Sequencing kit XLR70. Data processing and analysis was done using sequence editor SEED with external tools for sequence processing and analysis like MAFFT, mothur, UCLUST, USEARCH, or BLASTn and included pyrosequencing noise reduction (mothur v.1.27.0 [29]), chimerical sequences identification (UCLUST 3.0.167,[30]) and removal, sequence trimming to 550 bases, aligning (MAFFT 6.864b,[31]) and clustering (USEARCH 5.2.32,[30]) at 97% similarity to yield Operational Taxonomic Units (OTUs). Consensus sequences were constructed for species identification. Ribosomal Database Project [32] as well as BLASTn [33] hits against GenBank were used to generate best hits.

#### Detection Of Ureaplasma Species, Mycoplasma Hominis And Chlamydia Trachomatis And Other Bacteria In AF

DNA was isolated from AF using QIAamp DNA Mini Kit (Qiagen, Netherlands) according to the manufacturer’s instruction (protocol for isolation of bacterial DNA from biological fluids, elution in 100 μl). Multiplex real-time PCR was performed on a Rotor-Gene 6000 instrument (Qiagen, Netherlands) with the commercial kit AmpliSens C. trachomatis/Ureaplasma/M. hominis-FRT (Federal State Institution of Science, Central Research Institute of Epidemiology, Moscow, Russia) to detect DNA of *Ureaplasma* species, *Mycoplasma hominis*, and *Chlamydia trachomatis* simultaneously. A housekeeping gene assay for human beta-actin is included in the system, to examine the presence of inhibitors of the PCR reaction. Other bacteria in the AF have been identified by PCR targeting the 16S rRNA gene sequencing using following primers: 5´-AGGAGGTGATCCAACCGCA -3´, 5´-GGTTAAGTCCCGCAACGAGCGC-3´ as previously described [34]. Bacteria were identified using BLAST and SepsiTest-BLAST tools.

### Cervical fluid and AF IL-6 analyses

The cervical fluid concentration of IL-6 was assessed by the Human IL-6 Quantikine enzyme-linked immunosorbent assay (R&D Systems Inc., Minneapolis, MN, USA). The minimal detectable concentration was 0.70 pg/mL, and the interassay and intraassay coefficients were less than 10%. Absorbance values were measured at 450 nm using a Multiskan RC ELISA reader (Thermo Fisher Scientific, Waltham, MA, USA). AF IL-6 concentrations were assessed with a lateral flow immunoassay Milenia^®^ QuickLine IL-6 using Milenia^®^ POCScan Reader (Biotec GmbH, Giessen, Germany). The measurement range was 50–10000 pg/mL. Intra-lot- and inter-lot-variation was 12.1% and 15.5%, respectively.

### Diagnosis of MIAC

MIAC was determined based on a positive result of the PCR analysis for *Ureaplasma* species, *Mycoplasma hominis* and *Chlamydia trachomatis*, and/or by positive 16S rRNA gene amplification. Specific PCR for *Ureaplasma* spp., *Mycoplasma hominis/Chlamydia trachomatis* has a sensitivity of 50–100 copies/mL. The sensitivity of our method for detection bacterial 16S rRNA verified by DNA of *Streptococcus agalactiae*, *Streptococcus mutans* and *Haemophilus influenza* is 150–700 copies/mL. Sanger sequencing of 16S rRNA is only possible when the concentration of bacterial DNA in AF is at least 1500–7000 copies/mL.

### Diagnosis of histological chorioamnionitis

The degree of neutrophilic infiltration was evaluated separately in free membranes (amnion and chorion-decidua), chorionic plate and in the umbilical cord, according to the criteria provided by Salafia et al. [35]. A diagnosis of histological chorioamnionitis was made based on the presence of histological grades of chorion-decidua 3–4, chorionic plate 3–4, umbilical cord 1–4, and/or amnion 1–4 [35]. A single pathologist who was blinded to the clinical status of the patient performed the histopathological examinations.

### Diagnosis of IAI

IAI was defined as the presence of MIAC with AF IL-6 values higher than 1000 pg/mL [8].

### Statistical analyses

Demographic and clinical characteristics were compared using a nonparametric Jonckheere-Terpstra test for continuous variables and presented as medians (range). Categorical variables were compared using the Cochran-Armitage test for trends and the results were presented as numbers (%). For the analysis of the AF and cervical fluid IL-6 concentrations among the subgroups of women based on the presence and absence of MIAC with or without histological chorioamnionitis, a nonparametric Jonckheere-Terpstra test was used. Differences were considered statistically significant at *p*<0.05. All *p*-values were obtained from two-sided tests, and statistical analyses were performed using GraphPad Prism 5.03 for Mac OS X (GraphPad Software, San Diego, CA, USA), the SPSS 19.0 statistical package for Mac OS X (SPSS Inc., Chicago, IL, USA).

A heatmap of the relative bacterial abundance was created with R using the heatmap.2 package. Hierarchical clustering was performed using the Jensen-Shannon distance [36] as a distance measure and Ward's method minimizing the total within-cluster variation for linkage analysis [37]. These statistical analyses were performed using R version 3.1.0 [38].

## Results

### Demographic and clinical characteristics of the study population

In total, sixty-one women with a singleton pregnancy complicated by PPROM were included in the study. All women self-reported Caucasian race. The clinical and demographic characteristics are displayed in Table 1. Twelve women from the present study were part of our previous report on cervical fluid IL-6 and IL-8 levels [39].

**Table 1:** Maternal and neonatal characteristics of PPROM pregnancies according to community state type of cervical microbiota.

Characteristic	CST I (n = 25)	CST II (n = 13)	CST IV-A (n = 11)	CST IV-B (n = 12)	*p*-value
Maternal age [years, median (range)]	30 (21–41)	30 (17–35)	29 (18–36)	32 (18–37)	0.29
Pre-pregnancy body mass index [kg/m^2^, median (range)]	23.9 (17.6–31.0)	22.2 (19.1–33.5)	22.3 (16.5–32.9)	22.5 (18.4–34.3)	0.28
Primiparous [number (%)]	16 (64%)	6 (46%)	3 (27%)	5 (42%)	0.08
Smoking [number (%)]	3 (12%)	2 (15%)	3 (27%)	2 (17%)	0.48
Gestational age at admission [weeks+days, median (range)]	33+0 (25+2–36+3)	31+6 (24+2–36+4)	31+6 (24+1–35+0)	31+0 (28+5–35+6)	0.16
Gestational age at delivery [weeks, median (range)]	33+3 (25+2–36+4)	32+1 (24+5–36+4)	32+2 (24+4–35+0)	31+1 (28+5–35+6)	0.10
Latency from admission to delivery [hours, median (range)]	40 (5–150)	71 (7–224)	59 (16–150)	45 (7–102)	0.87
Microbial invasion of the amniotic cavity [number (%)]	4 (16%)	3 (23%)	4 (36%)	6 (50%)	**0.02**
Cervical fluid IL-6 [pg/mL, median (range)]	145 (16–609)	166 (15–661)	420 (37–550)	322 (65–562)	**0.004**
CRP levels at admission [mg/L, median (range)]	7.6 (2.1–31.1)	6.1 (1.5–41.0)	6.5 (1.0–21.7)	6.4 (1.7–68.5)	0.38
WBC count ad admission [x10^9^ L, median (range)]	12.0 (7.4–20.6)	11.9 (7.9–29.0)	11.6 (9.3–16.8)	14.0 (7.4–19.5)	0.85
Spontaneous vaginal delivery [number (%)]	16 (64%)	7 (54%)	7 (64%)	10 (83%)	0.30
Birth weight [grams, median (range)]	2060 (780–3190)	1930 (650–2450)	1890 (670–2280)	1725 (1210–2570)	0.06
Histological chorioamnionitis [number (%)]	17 (68%)	9 (69%)	8 (73%)	9 (75%)	0.64
Apgar score—5 minutes [median (range)]	10 (8–10)	9 (1–10)	9 (0–10)	10 (8–10)	0.60
Apgar score—10 minutes [median (range)]	10 (8–10)	10 (1–10)	10 (0–10)	10 (9–10)	0.45

Abbreviations:

PPROM: Preterm prelabor rupture of membranes

IL-6: Interleukin-6

CRP: C-reactive protein

WBC: White blood cells

Continuous variables were compared using a nonparametric Jonckheere-Terpstra test. Categorical variables were compared using either Cochran-Armitage test for trend test. Statistically significant results are marked in bold.

### Characterization of the microbial taxa in the cervical fluid

The cervical microbiota was characterized using massively parallel sequencing of 16S rRNA genes. The dataset consisted of 79,194 high-quality sequences of 550bp. The average number of sequence per sample was 1365 (95% confidence interval 1172–1558). Taxonomic assignment of the sequences identified 227 taxa in the cervical microbiota. Taxonomic assignments of cervical microbiota are shown in S1 Table.

### The cervical microbiota in women with PPROM

To study the cervical bacterial communities in women with PPROM, the vectors of relative abundance of bacterial taxa were hierarchically clustered using the Jensen-Shannon divergence metric and Ward linkage. The overall diversity measured as Shannon-Wiener diversity index in our study (median 1.5; range 0.4–3.4) was similar to the results published by Ravel et at. [21] Satisfactory depth of sequencing was validated by rarefaction curves (data not shown).

**Fig 1:**
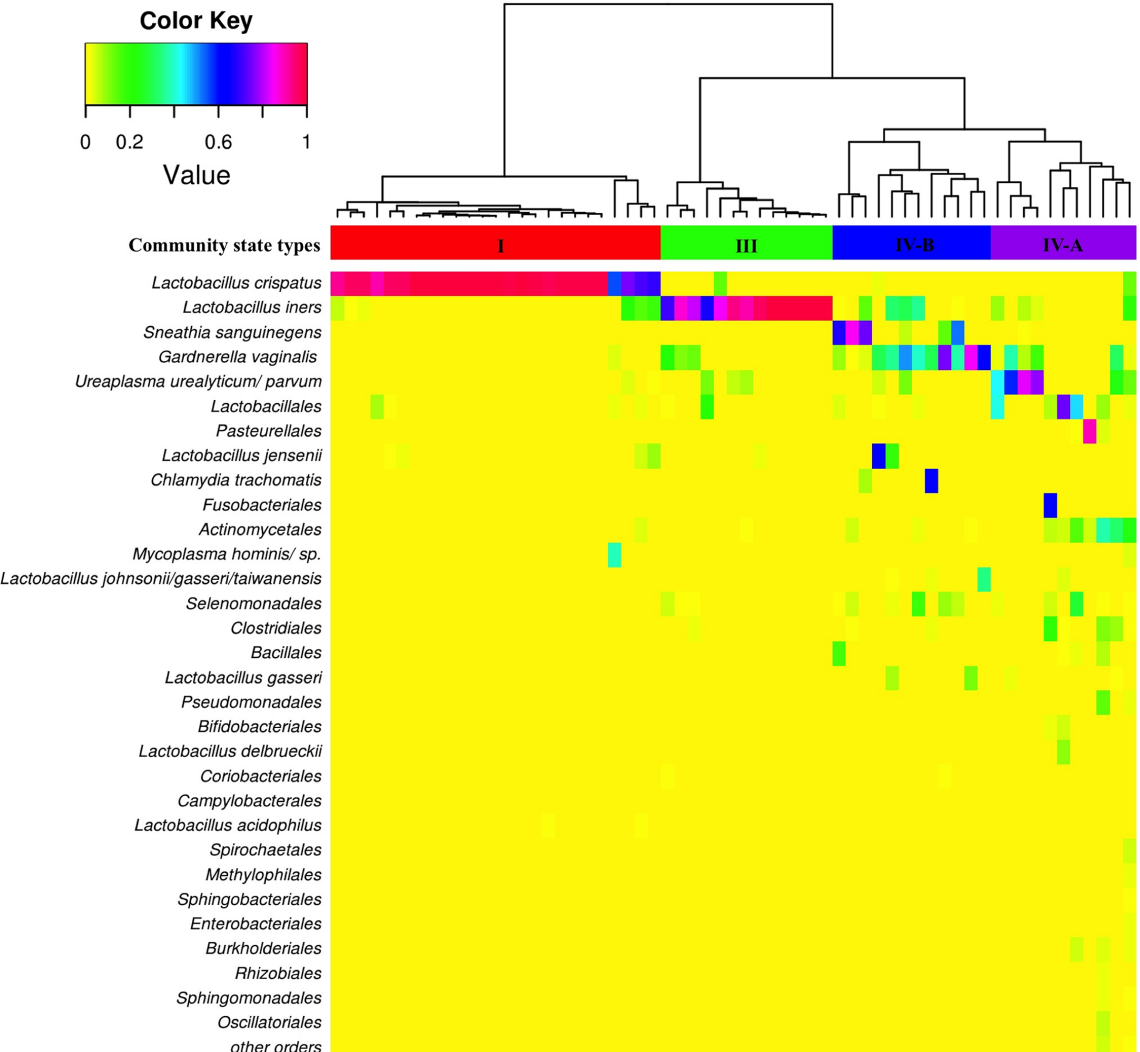
Heatmap of percentage abundance of selected species and remaining orders of the cervical microbiome of sixty-one women with PPROM. Ward linkage hierarchical clustering of Jensen-Shannon metrics identified four community state types (CST I, III, IV-A, and IV-B). The upper color bar shows the four community state types.

**Fig 2:**
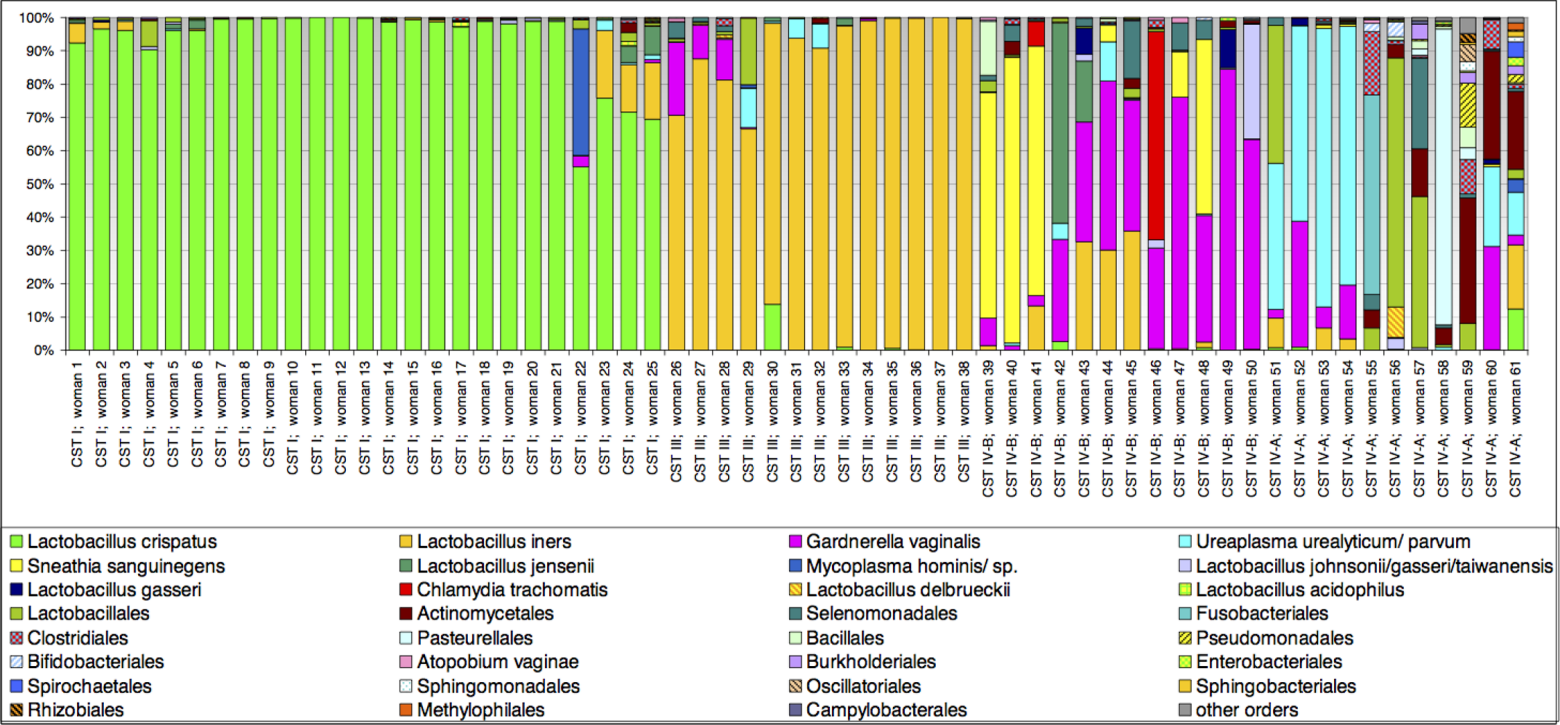
Bar chart of percentage abundance of selected species and remaining orders of bacteria in the cervical microbiota of sixty-one women with PPROM.

Community states were clustered into four CSTs with similar bacterial compositions and abundance (Figs 1 and 2). CST I (*Lactobacillus crispatus* dominated) and CST III (*Lactobacillus iners* dominated) were found in 41% (25/61) and 21% (13/61) of women, respectively. Communities that were clustered into the CST IV-A or IV-B did not have *Lactobacillus* species as the most abundant bacteria (except for one woman [No. 42] who had *Lactobacillus jensenii* as the most dominant bacteria) and differed in species composition (Figs 1 and 2). CST IV-A and CST IV-B were identified in 18% (11/61) and 20% (12/61) of women, respectively. *Ureaplasma* species, *Propionibacterium acnes*, *Fusobacterium nucleatum*, *Veillonela* species, *Streptococcus* species, and *Haemophilus influenzae* dominated the bacterial communities of CST IV-A. In contrast, the CST IV-B was characterized by the high abundance of *Gardnerella vaginalis* and/or *Sneathia sanguinegens* and other species previously shown to be associated with bacterial vaginosis [40,41].

### Cervical fluid IL-6 concentrations according to the CSTs

**Fig 3:**
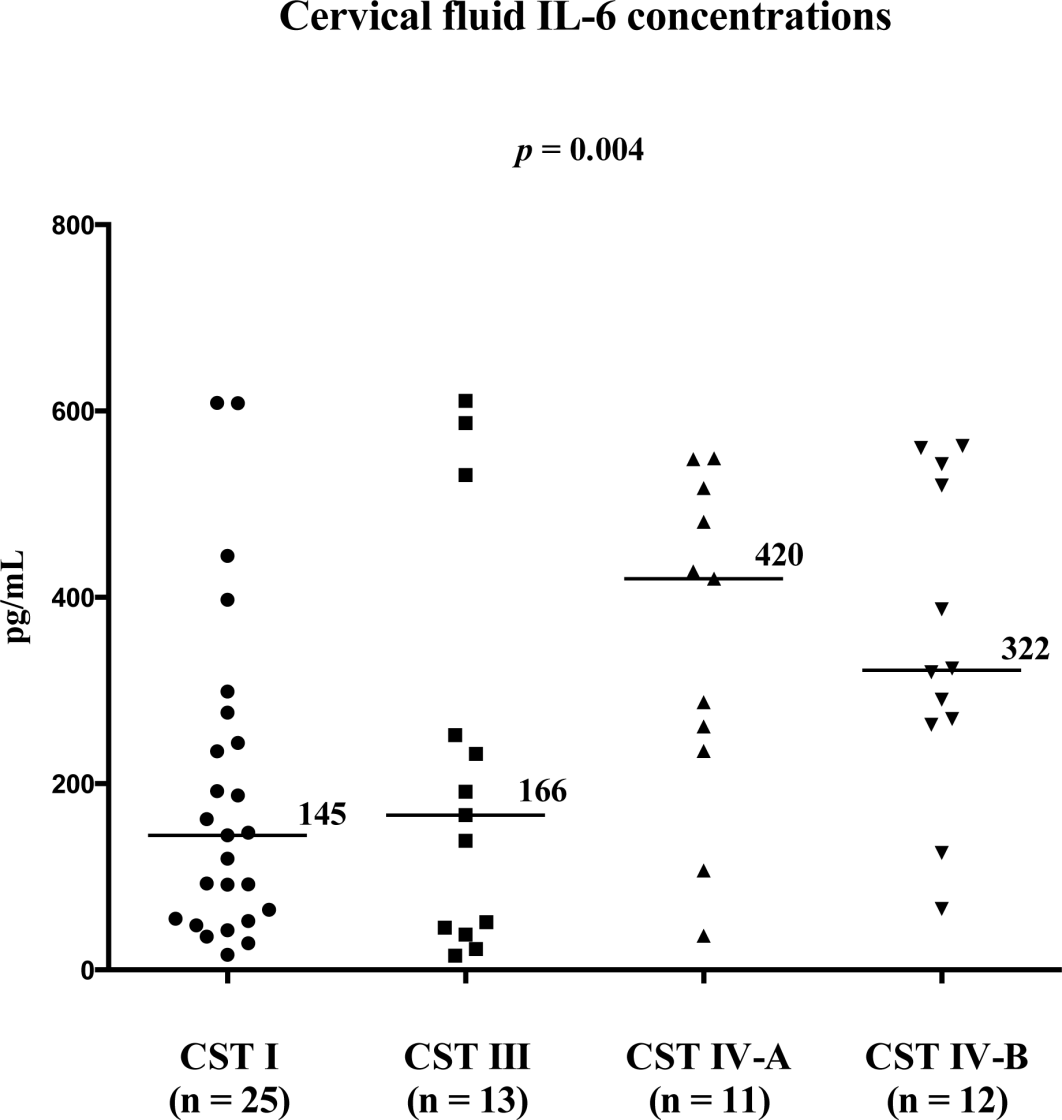
The cervical fluid IL-6 concentration levels in PPROM pregnancies according to cervical bacteria CSTs. Abbreviations: IL-6—interleukin-6; PPROM—preterm prelabor rupture of membranes; CST—community state type.

To analyze differences in cervical inflammatory response with respect to cervical microbiota, cervical fluid IL-6 concentrations were evaluated. There was a difference in cervical fluid IL-6 concentrations among women with CST I, III, IV-A, and IV-B (CST I: median 145 pg/mL, range 16–609 pg/mL; CST III: median 166 pg/mL, range 15–611 pg/mL; CST IV-A: median 420 pg/mL, range 37–549; CST IV-B: median 322 pg/mL, range 65–562 pg/mL; *p* = 0.004) (Fig 3). Women with non-*Lactobacillus* dominated CSTs (IV-A and IV-B) had higher cervical fluid IL-6 concentrations than women with *Lactobacillus* dominated CSTs (I and III) (non-*Lactobacillus* CSTs: median 324 pg/mL, range 37–562 vs. *Lactobacillus* CSTs: median 149 pg/mL, range 37–562; *p* = 0.002).

### MIAC according to the CSTs

**Fig 4:**
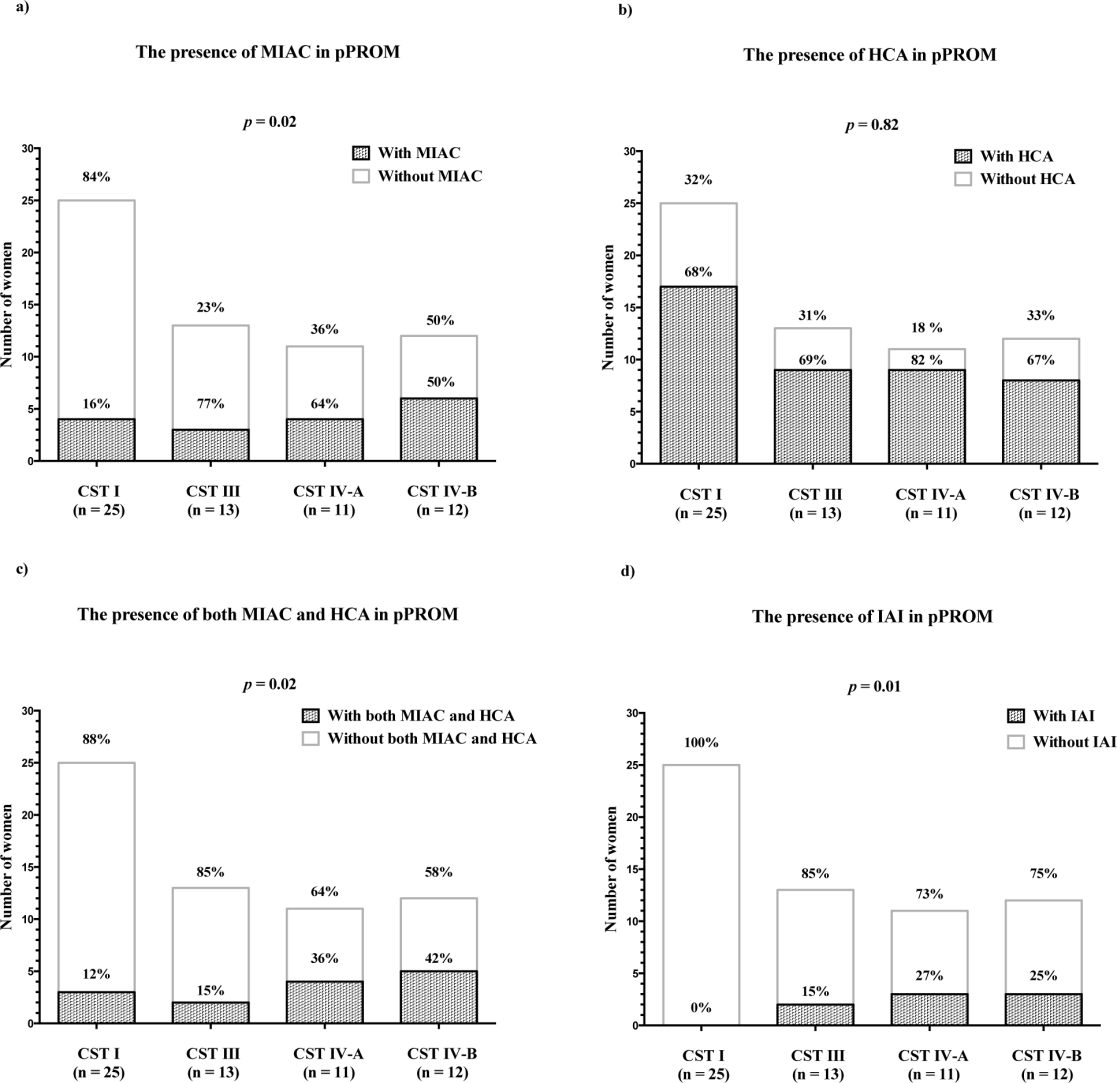
The presence of MIAC (a), histological chorioamnionitis (b), both MIAC and histological chorioamnionitis (c), and IAI (d) in PPROM with respect to cervical bacteria CSTs. Abbreviations: MIAC—microbial invasion of the amniotic cavity; IAI—intraamniotic infection;—preterm prelabor rupture of membranes; CST—community state type.

**Fig 5:**
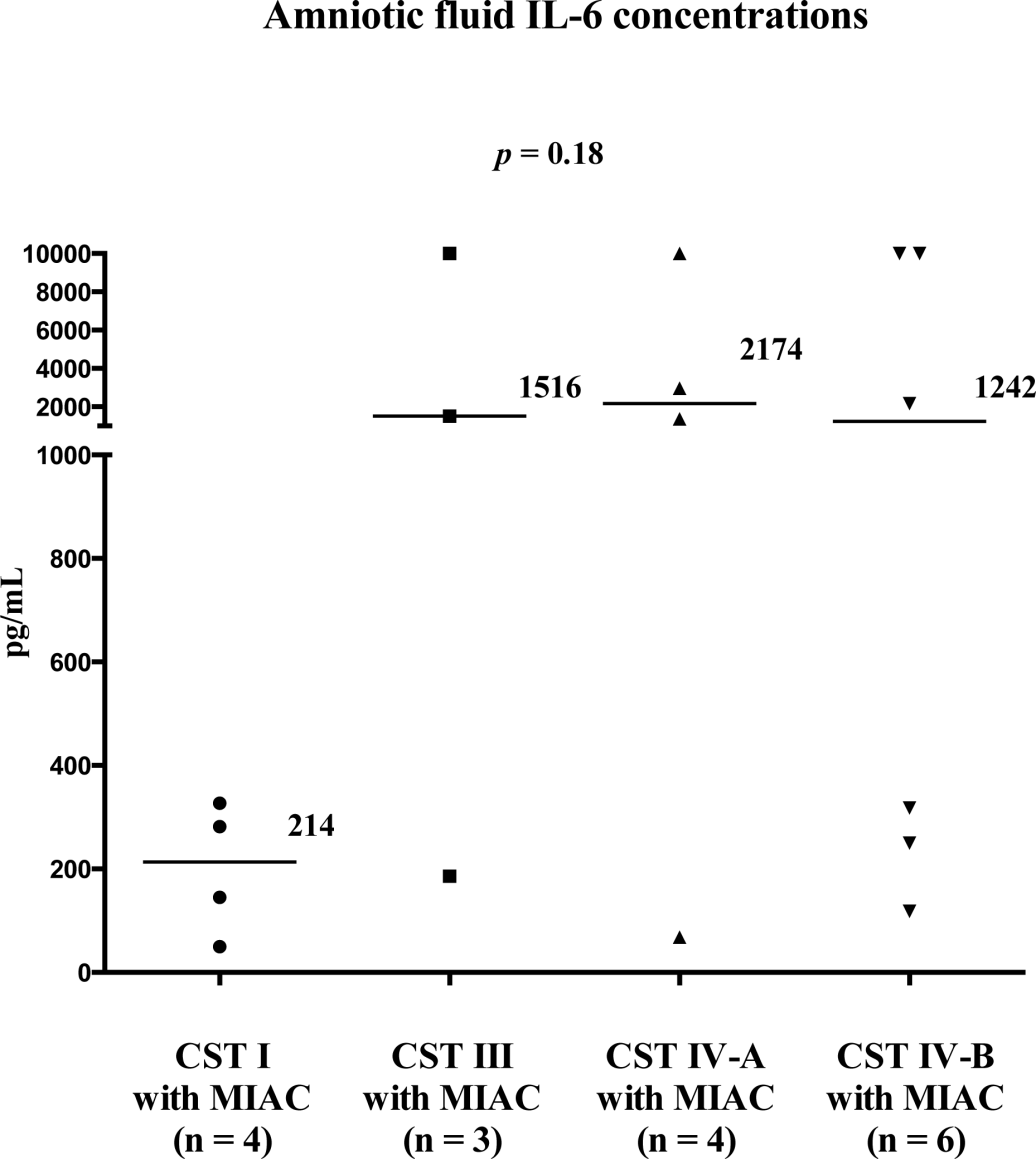
Amniotic fluid IL-6 concentrations in women with PPROM complicated by MIAC with respect to cervical bacteria CST. Abbreviations: IL-6—interleukin-6; MIAC—microbial invasion of the amniotic cavity; PPROM—preterm prelabor rupture of membranes; CST—community state type.

A difference was observed in the incidence of MIAC among women in different CST groups. MIAC was present in women with CST I in 16% (4/25), CST III in 23% (3/13), CST IV-A in 36% (4/11), and CST IV-B in 50% (6/12) (*p* = 0.02; Fig 4A). All bacteria identified in the AF, along with the most abundant bacteria in the cervical fluid, are shown in Table 2. To determine whether the intensity of MIAC-associated intraamniotic inflammatory response varied among CSTs, AF IL-6 concentrations in women with MIAC were evaluated. There was no significant difference in AF IL-6 among CSTs (CST I: median 214 pg/mL [50–327]; CST III: median 1516 pg/mL [186–10000]; CST IV-A: median 2174 pg/mL [68–10000] and CST IV-B: median 1242 pg/mL [118–10000]); (*p* = 0.18; Fig 5). Nevertheless, women with MIAC in CST I had very low AF IL-6 concentrations. Moreover, the microbial load of bacteria found in the AF of women with CST I was very low (Fig 5 and Table 2). Accordingly, MIAC in women with CST I appeared to be due to colonization alone.

**Table 2:** Bacteria identified in the amniotic fluid of pregnancies complicated by PPROM.

Woman No.	CST No.	Bacteria identified in the amniotic fluid	Dominant bacteria in the cervical fluid
5	I	*Ureaplasma* spp.	*Lactobacillus crispatus*
8	I	*Ureaplasma* spp.; *Chlamydia trachomatis*	*Lactobacillus crispatus*
14	I	*Ureaplasma* spp.	*Lactobacillus crispatus*
23	I	*Ureaplasma* spp.	*Lactobacillus crispatus*
27	III	*Ureaplasma* spp.; *Chlamydia trachomatis*	*Lactobacillus iners*
28	III	*Chlamydia trachomatis*	*Lactobacillus iners*
29	III	*Ureaplasma* spp.; *Mycoplasma hominis*	*Lactobacillus iners*
39	IV-B	*Ureaplasma* spp.; *Mycoplasma hominis*	*Sneathia sanguinegens*
40	IV-B	*Ureaplasma* spp.; *Leptotrichia amnionii*	*Sneathia sanguinegens*
41	IV-B	*Chlamydia trachomatis*	*Sneathia sanguinegens*
42	IV-B	*Ureaplasma* spp.	*Lactobacillus jensenii*
46	IV-B	*Chlamydia trachomatis*	*Chlamydia trachomatis*
48	IV-B	*Ureaplasma* spp.; *Chlamydia trachomatis*	*Sneathia sanguinegens*
51	IV-A	*Ureaplasma* spp.	*Ureaplasma urealyticum*
53	IV-A	*Ureaplasma* spp.	*Ureaplasma urealyticum*
58	IV-A	*Haemophilus influenzae*	*Haemophilus influenzae*
61	IV-A	*Mycoplasma hominis*; *Leptotrichia amnionii*	*Propionibacterium acnes*

Abbreviations:

PPROM: Preterm prelabor rupture of membranes

CST: Community state type

### Histological chorioamnionitis according to CSTs

No difference was found in the incidence of histological chorioamnionitis among women in CSTs I, III, IV-A, and IV-B groups. In women with CST I 68% (17/25) had MIAC, 69% (3/13) in CST III, 81% (9/11) in CST IV-A and 67% (8/12) in CST IV-B (*p* = 0.82; Fig 4B). The presence of both MIAC and histological chorioamnionitis was significantly different between the CSTs. Women with CST I had both MIAC and histological chorioamnionitis in 12% (3/25), CST III in 15% (2/13), CST IV-A in 36% (4/11) and CTS IV-B in 41% (5/12) (*p* = 0.02; Fig 4C).

### IAI according to CSTs

The incidence of IAI among the CST groups varied significantly. In women with CST I, none had IAI (0/25), with CST III 15% (2/13) had IAI, with CST IV-A 27% (3/11), and with CST IV-B 25% (3/12) (*p* = 0.01; Fig 4D).

## Discussion

The principal findings of this study were as follows: i) four CSTs (I, III, IV-A, and IV-B) were observed in women with PPROM; ii) the local cervical inflammatory response, characterized by cervical fluid IL-6 concentration, varied among CSTs; iii) the incidence and composition of MIAC varied among CSTs; iv) no difference in the rate of histological chorioamnionitis among CSTs was found; v) CST IV-B was associated with the highest incidence of MIAC; vi) CST I (*L*. *crispatus* dominated) represented the dominant type of PPROM with a low rate of MIAC characterized by low microbial load of bacteria and low intraamniotic inflammatory response. In addition, no IAI was found in this CST group; vii) CST IV-A and IV-B were associated with the highest incidence of IAI and the presence of both MIAC and histological chorioamnionitis.

Four of six previously described CSTs were identified in this study and CST I was the most common (41%). The incidence of CSTs I, III, and IV in women with PPROM was different compared to previously described rates in women with uncomplicated pregnancies and with a spontaneous preterm birth [26]. Women with PPROM had a lower incidence of CST III and a higher incidence of CSTs I and IV. In concordance with the previous report, we did not find CST II or V (dominated by *Lactobacillus gasseri* and *Lactobacillus jensenii*, respectively) [26]. In our study, the distribution of CSTs among women with PPROM was similar to the distribution of CSTs in white non-pregnant women compared to women with uncomplicated pregnancies and women who had a spontaneous preterm delivery before 34 weeks [20–22,26]. This may be a population and region specific observation since the women in our study were Caucasian and from the eastern part of the Czech Republic.

The local inflammatory response in the lower genital tract is mediated through epithelial cells, which are in direct contact with bacteria, and represent the entry point for bacteria invading the upper genital tract [41]. A recent study demonstrated that endocervical epithelial cells produce a higher inflammatory response compared to ectocervical and vaginal epithelial cells [41]. To test whether there was a difference in cervical inflammatory response among CSTs, we evaluated the concentration of IL-6, an inflammatory pleiotropic cytokine in the cervical fluid. Women with non-*Lactobacillus* dominated CSTs (CST IV-A and IV-B) had approximately 2–2.5 fold higher cervical fluid IL-6 concentrations than *Lactobacillus* dominated CSTs (CST I and III). This means that non-*Lactobacillus* dominated CSTs are associated with a stronger cervical inflammatory response which is concordant with previous findings that bacterial vaginosis associated bacteria are able to elicit a stronger cytokine response from epithelial cells of the lower genital tract than commensal strains of *Lactobacilli* [41].

The majority of bacteria found in AF probably gain access through ascending colonization from the lower genital tract [42–46]. However, bacteria have to penetrate the cervical mucous barrier and cross the fetal membranes to reach the AF. A recent study has demonstrated that ascending colonization of bacteria from the lower genital tract to the maternal-fetal interface is limited in pregnancy due to the antimicrobial properties of cervical mucus [12]. Furthermore, viral infection of the cervix decreases the protective properties of the cervix and permits the migration of bacteria from the lower genital tract [12]. Our study revealed that the incidence of MIAC alone, the presence of both MIAC and histological chorioamnionitis, and IAI varied among different CSTs. Women with non-*Lactobacillus* dominated CSTs had the highest rates of these complications. Moreover, women with CTS IV-B had the highest incidence of MIAC. Both of the bacteria (*Gardnerella vaginalis* and *Sneathia sanguinegens*), which are dominant in CST IV-B, are bacterial vaginosis associated bacteria [40,47]. In addition, all women who had *Sneathia sanguinegens* as the dominant bacteria had MIAC. Despite only three cases, this finding should attract further investigation. We can speculate whether the bacterial composition of cervical microbiota or the concurrent presence of viral infection of the cervix is responsible for the high rate of MIAC. It is well documented that bacterial vaginosis is a condition commonly associated with human papillomavirus infection of the cervix [48,49].

In this study, 70% of PPROM pregnancies were complicated by histological chorioamnionitis. Nevertheless, histological chorioamnionitis is a heterogeneous entity, which can be divided in two subgroups (infectious and non-infectious) based upon the presence or absence of MIAC. We have demonstrated that the combination of MIAC and histological chorioamnionitis represents a subgroup of PPROM with the strongest intraamniotic and fetal inflammatory response [50,51]. Interestingly, a low rate of infectious histological chorioamnionitis (with MIAC) was found in both *Lactobacillus* dominated CSTs which is in contrast to a high rate in both non-*Lactobacillus* dominated CSTs.

A recent study has demonstrated that the presence of *L*. *crispatus* as the dominant bacteria of the lower genital tract microbiota prevents lower genital tract colonization by *E*. *coli* [52]. In our study, women with *L*. *crispatus* dominant cervical flora (CST I) had the lowest incidence of MIAC, of both MIAC and histological chorioamnionitis and no evidence of IAI. In addition, the bacterial load of *Ureaplasma* species found in the AF of women with CST I was low (data not shown). This is an important observation because the most abundant bacteria in the cervix can help clinicians to anticipate the risk of MIAC and IAI without the need to perform an invasive procedure such as amniocentesis. The risk of IAI and inflammation appears to be very low when *L*. *crispatus* is the most abundant cervical bacterium. On the other hand, the presence of non-*Lactobacillus* bacteria as the dominant cervical bacteria might predict women who are at higher risk of MIAC and IAI. Identification of the most dominant cervical bacteria in clinical practice would not require expensive high-throughput techniques, since specific real time PCR assays can be used. Based on this study, we propose that a microbiome signature of the cervix may act as a biomarker for the risk of MIAC, IAI and histological chorioamnionitis following PPROM.

The unique aspect of this study is that samples of cervical and AF were obtained at the same time on admission before administration of antibiotics and corticosteroids. This approach gave us an excellent opportunity to evaluate and characterize cervical microbiota with respect to local inflammatory response and MIAC. However, our study has some limitation. In a healthy term pregnancy, AF has antimicrobial properties and can inhibit the growth of bacteria such as *Escherichia coli*, *Staphylococcus aureus*, *Streptococcus agalactiae*, *Streptococcus faecalis* [53–56]. In PPROM, AF leakage can affect the cervical microbiome and inflammatory cytokine pool. This is an unavoidable confounder when studying cervical microbiota and inflammation in PPROM and in case only designs. Due to changes in the AF proteome during pregnancy and the variable duration of exposure to AF, it is not clear what effect PPROM will have on the cervical microbiota [57]. Therefore, we suggest caution when interpreting the data presented in this manuscript. These can be partially addressed by using cervical samples from gestational age matched spontaneous preterm birth with intact membranes with similar MIAC and histological chorioamnionitis status. Regardless, it is unlikely that a correlation between CST IV-A and CST IV-B and inflammation and lack thereof with other groups are mere coincidences and we argue that this pattern needs further verification and validation in independent cohorts. Second, our hypothesis was that following PPROM, different CSTs influence the likelihood of MIAC with or without histological chorioamnionitis, the cervical inflammatory response and IAI. Accordingly, we recognize that our sampling may not reflect the cervical microbiome prior to PPROM. Third, our analyses were based on a relatively small cohort so a type II error cannot be ruled out. Furthermore, MIAC was not characterized using next-generation sequencing technology but specific PCR for genital mycoplasmas and *Chlamydia trachomatis* and/or positive 16S rRNA gene amplification. We believe that in depth characterization of intraamniotic microbiota would be of interest but this was beyond the scope of the study.

## Supporting Information

S1 Table Tables and charts of microbiome composition and diversity indices in individual women for clinically relevant taxa and basic species level. (XLS)
